# Fire Resistance and Colorimetric Analysis of Lightweight Fiber-Reinforced Foamed Alkali-Activated Hybrid Binders

**DOI:** 10.3390/ma18214829

**Published:** 2025-10-22

**Authors:** Magdalena Rudziewicz, Katarzyna Mróz, Marcin Maroszek, Paweł Wołkanowski, Marek Hebda

**Affiliations:** 1 Faculty of Materials Engineering and Physics, Cracow University of Technology, Warszawska 24, 31-155 Kraków, Poland; magdalena.rudziewicz@doktorant.pk.edu.pl (M.R.); marcin.maroszek@doktorant.pk.edu.pl (M.M.); 2 Chair of Building Materials Engineering, Faculty of Civil Engineering, Cracow University of Technology, Warszawska 24, 31-155 Kraków, Poland; katarzyna.mroz@pk.edu.pl; 3 Faculty of Mechanical Engineering, Cracow University of Technology, Warszawska 24, 31-155 Kraków, Poland; pawel.wolkanowski@pk.edu.pl

**Keywords:** alkali-activated hybrid binder foams, merino wool, basalt fibers, polypropylene fibers, coconut fibers, fire resistance, pore structure analysis

## Abstract

In response to escalating environmental concerns, the construction industry is under growing pressure to adopt sustainable practices. As a major consumer of natural resources and a significant emitter of greenhouse gases, it paradoxically holds the potential to become a leader in green transformation. This study investigates the development of innovative, fire-resistant, and alkali-activated hybrid binder foams incorporating recycled materials: fly ash, coal slag, and ground brick waste, as sustainable alternatives to traditional building materials. The fire resistance performance at a technical scale and the thermal behavior of fiber-reinforced, alkali-activated hybrid binder foams synthesized from recycled aluminosilicate precursors were determined. The properties of unreinforced composite were compared with the composites reinforced with merino wool, basalt fibers, polypropylene fibers, and coconut fiber. Small-scale fire-resistance tests revealed that merino wool-reinforced composites exhibited the best thermal insulation performance, maintaining structural integrity, that is, retaining shape and continuity without delamination or collapse for 83 min under fire exposure. Analyses combining chemical characterization (X-ray fluorescence) with microstructural methods (computed tomography and colorimetry) confirmed that fire performance is strongly influenced not only by fiber type but also by pore distribution, phase composition, and oxide migration under thermal loading. These findings demonstrate the potential of fiber-reinforced foamed, alkali-activated hybrid binder as eco-efficient, printable materials for fire-safe and thermally demanding construction applications.

## 1. Introduction

In light of increasingly stringent fire safety regulations, the thermal resistance of construction materials has become a parameter of paramount importance. Concrete, although traditionally regarded as inherently fire-resistant, exhibits highly complex and not yet fully elucidated behavior under real fire exposure [1,2,3]. Elevated temperatures induce profound physicochemical transformations within both the cementitious matrix and the aggregate phase [4,5]. Fire exposure during a structure’s service life poses a serious risk to durability and occupant safety, making fire resistance a key criterion in material evaluation [6]. Although concrete is often assumed to be fire-resistant, elevated temperatures degrade its internal structure, leading to cracking, spalling, and accelerated propagation of pre-existing microcracks, which significantly reduces mechanical performance with strength losses approaching 95% at ~1000 °C and may, under critical conditions, endanger structural stability, integrity, and human life [7]. In addition to these performance limitations, the production of conventional concrete requires intensive raw material extraction and generates significant environmental impacts [8,9,10,11]. To address these issues, research has increasingly focused on sustainable and thermally robust alternatives, with geopolymer-based hybrid concretes emerging as promising substitutes for Portland cement systems [12]. Synthesized from aluminosilicate-rich industrial by-products [13,14,15,16], this material offers both a reduced environmental footprint [17,18,19,20,21], and enhanced resistance to elevated temperatures, reportedly maintaining structural integrity at temperatures of 1000–1200 °C [22,23]. Fire resistance in geopolymer concrete can be further improved through the incorporation of functional additives, including various types of fibers (e.g., carbon [24], steel [25], glass [26], basalt [27,28], polypropylene [29]), or other additives, such as marble powder [30], recycled glass [31], graphene nanoplatelets [32] and recycled rubber particles [33]. Furthermore, experimental investigations have shown that a 20 mm layer of geopolymer foam applied to a polystyrene substrate can withstand exposure to temperatures of up to 800 °C for a minimum duration of 1000 s [28].

### 1.1. Fire-Resistant and Reaction-Foamed Geopolymer Composites

Despite significant advancements in geopolymer research over the past decades, several challenges still persist with regard to their practical application. While thermal curing can enhance the mechanical and durability properties of geopolymers, numerous studies have shown that satisfactory performance can also be achieved under ambient conditions, depending on the chemistry and proportions of the precursors. To address this constraint, Mejia et al. [34] proposed a novel class of materials, so-called hybrid geopolymers, that combine traditional aluminosilicate geopolymers with calcium-rich components such as ground granulated blast furnace slag or ordinary Portland cement (OPC). This approach facilitates effective ambient-temperature curing, thereby enabling broader adoption of geopolymer technologies in engineering practice. The production of geopolymer composites based on industrial and construction waste materials, including fly ash, bottom ash, and ground construction brick debris [35,36], not only significantly reduces landfill waste but also mitigates the demand for traditional energy-intensive cementitious binders. Ground demolition brick (GB), owing to its mineral composition and prior calcination at temperatures of 900–1000 °C, exhibits exceptional thermal resistance, making it a particularly advantageous component for geopolymer composites intended for fire-exposed applications. Hybrid foamed geopolymers incorporating GB are characterized by their low density and limited thermal conductivity, making them effective thermal insulators [37,38,39]. A notable example of such materials is foamed geopolymer composite, which possesses high total porosity and demonstrates a remarkable fire resistance. foamed geopolymer composite has achieved Euroclass A1 classification, indicating non-combustibility and the absence of toxic gas emissions under fire conditions [40]. Experimental investigations have demonstrated that GB-based geopolymers retain approximately 75–85% of their initial compressive strength following exposure to 800 °C, whereas Portland cement concrete under comparable conditions may lose more than 90% of its load-bearing capacity [41]. Other study reports that after exposure to 600 °C the compressive strength of high-performance concrete decreased, and upon subsequent treatment (water or cyclic curing) partial recovery of strength was observed [42]. Further reported that although GB-based geopolymers exhibit mass and density losses of ~8% at 600 °C and ~10% at 800 °C, these changes exert negligible influence on their overall structural integrity. Consistent with conventional geopolymers, GB-based foamed systems exhibit high water vapor permeability, which underpins their combined fire resistance and thermal insulation capacity. Łach et al. [43] confirmed this behavior, showing that the reverse side of a geopolymer panel remained below 100 °C despite the exposed surface reaching 1100 °C.

The majority of reported studies on foamed geopolymer composites employ aluminum powder or hydrogen peroxide as foaming agents, both of which effectively generate porosity and thereby enhance thermal insulation. In alkaline solution, aluminum reacts to release hydrogen gas, while hydrogen peroxide decomposes to oxygen, facilitating pore formation; these mechanisms were detailed in our previous work [44]. Both methods are considered highly effective for producing materials with desirable properties, such as improved high-temperature resistance and thermal insulation. However, research on synthetic foaming agents in staged (pre-foamed) processes remains limited, despite their potential to improve control of material properties and enable practical application in on-site construction, particularly for emerging 3D printing technologies. A study conducted by Anggarini et al. [45] demonstrated that pre-foamed materials exhibited superior thermal resistance and fewer surface defects relative to conventional methods. This approach promotes the formation of homogeneous porosity and lightweight composites with enhanced thermal insulation, without the reliance on highly reactive foaming agents such as aluminum powder.

### 1.2. Fiber-Reinforced Geopolymer-Based Materials

Despite advantages such as reduced CO_2_ emissions, chemical resistance, and improved fire protection, geopolymer composites are generally more brittle than conventional concrete, particularly after fire exposure [46,47]. Current research therefore emphasizes fiber incorporation into geopolymer matrices [48] to mitigate brittleness and further enhance fire resistance. Fiber reinforcement is considered one of the most effective methods for reducing the inherent brittleness of foamed geopolymer composites. These materials are particularly susceptible to microcracking and sudden, brittle failure, limiting their suitability for structural applications subjected to dynamic or impact loads. The inclusion of fibers substantially improves the mechanical performance of the matrix, thereby addressing this limitation [48]. Fibers primarily serve to bridge microcracks, thereby preventing their further propagation, a mechanism often referred to as the ‘bridging effect’ [49]. Within the composite structure, fibers redistribute stress from weakened zones to undamaged regions, promoting a more uniform stress distribution and enhancing the system’s overall ductility.

Among fibers used in geopolymer composites, steel fibers are especially effective in enhancing ductility and crack resistance. With a high elastic modulus (~210 GPa) and tensile strength above 2500 MPa, they improve mechanical performance and limit crack propagation. Hooked-end types further increase pull-out resistance through mechanical anchorage, strengthening the fiber–matrix bond under load [50,51,52,53,54,55,56].

Polyvinyl alcohol (PVA) fibers are highly effective due to their hydrophilic nature and strong adhesion to the geopolymer matrix, enabling efficient crack bridging and improved mechanical performance. PVA reinforcement has been shown to increase flexural deflection up to 50-fold, greatly enhancing deformation capacity, energy absorption, and shifting material behavior from brittle to quasi-ductile [57,58,59,60,61,62].

Natural fibers, such as sisal, hemp, coconut, and flax, represent a more environmentally sustainable alternative. While their influence on mechanical properties is generally positive, it is often less pronounced compared to steel or PVA fibers. Studies [63,64] have shown that the addition of coconut fibers may provide an increase in compressive strength by up to 25%, while sisal fibers contribute approximately 6%. However, natural fibers often exhibit lower durability in alkaline environments and weaker matrix bonding, which may reduce composite strength at higher fiber contents.

Basalt, glass, and carbon fibers also play a significant role in enhancing the performance of geopolymers, particularly under elevated temperature and chemically aggressive conditions. Basalt fibers offer excellent thermal stability, with melting points exceeding 1000 °C [65]. Their incorporation into the hybrid geopolymer matrix has been shown to improve thermal resistance, as evidenced by reduced strength losses after high-temperature exposure [66,67]. While somewhat more brittle than steel or PVA fibers, basalt fibers still significantly improve structural integrity under extreme conditions. Carbon fibers, due to their distinct chemical and physical characteristics, also enhance integrity at elevated temperatures; their high thermal conductivity promotes uniform heat dissipation, further improving fire resistance [68,69,70]. Glass fibers are another widely used reinforcement, valued for increased tensile strength and cost efficiency. Their amorphous structure and Young’s modulus, comparable to bulk glass, increase stiffness and limit deformability, while also providing favorable thermal insulation. Strong interfacial adhesion between glass fibers and the geopolymer matrix has been observed before and after exposure to 600 °C, indicating preservation of composite integrity under thermal stress [71]. Furthermore, improved aging resistance has been reported in glass-fiber-reinforced geopolymer composites when compared to unreinforced systems, suggesting an enhancement in long-term mechanical performance.

Among the most extensively studied fibers with respect to fire resistance are polypropylene (PP) fibers. Their primary advantage lies in their relatively low melting point (approximately 160–170 °C), which results in the formation of microchannels within the composite structure upon thermal exposure [72]. These microchannels serve as pathways for water vapor release, thereby relieving internal pressure and significantly reducing the risk of fire spalling in high-tightness materials, such as high-performance concrete (HPC) [73].

Another promising strategy for improving thermal stability involves the development of hybrid fiber-reinforced systems incorporating nanostructured additives. Previous investigations have reported on geopolymer matrices modified with graphene nanoplatelets (GNPs) and crumb rubber (CR), the latter of which is a recycled elastomeric by-product [32,33]. The GNPs serve to enhance both thermal conductivity and structural reinforcement within the matrix, while CR contributes thermal insulating properties and facilitates dissipation of thermally induced stresses. In parallel, the integration of mineral wool fibers into geopolymer composites has emerged as a viable alternative to conventional fire-resistant materials due to the synergy of mechanical strength, thermal insulation, and fire-resistant characteristics [74]. Murri et al. [75] reported that a composite containing 23% of mineral wool fibers achieved fire reaction classification A2 under the UNI-EN 13501-1 standard, categorizing it as non-combustible with limited contribution to fire development. It is important to note, however, that the fire performance of foamed geopolymer composites is not invariant, but is instead highly dependent on a range of factors, including precursor composition, foam volume fraction, type and granulometry of aggregates, calcium content in the aluminosilicate source materials, as well as the type and molarity of the alkaline activator solution. In addition, the type, morphology, and dosage of functional additives may exert a significant influence on thermal performance. Accordingly, continued investigations into these parameters are critical for optimizing material properties to meet application-specific requirements, particularly in construction scenarios involving high thermal loads.

A detailed market analysis confirms the rapidly growing demand for geopolymeric materials, particularly foamed geopolymers and their composites, which are increasingly recognized as sustainable alternatives in construction. Owing to their high thermal resistance, low environmental footprint, and adaptability to various climatic conditions, foamed geopolymers have considerable potential as insulating materials. The present research builds upon these premises and introduces significant academic innovations. Firstly, it demonstrates the development of fiber-reinforced foamed hybrid geopolymers suitable for additive manufacturing, enabling the 3D printing of geometrically complex, fire-resistant, and thermally insulating components. Secondly, the study highlights the novel capability of using such composites to monitor and control temperature distribution in critical and hard-to-access infrastructure, such as tunnels, where conventional concrete panels show limited durability under fire exposure. By integrating recycled aluminosilicate-rich raw materials with fiber reinforcement and adapting formulations for 3D printing, this work expands the scope of geopolymer applications from sustainable construction to advanced safety-critical systems, positioning these materials as promising solutions for both environmental responsibility and fire resilience in the built environment.

## 2. Materials and Methods

### 2.1. Materials

**Figure 1:**
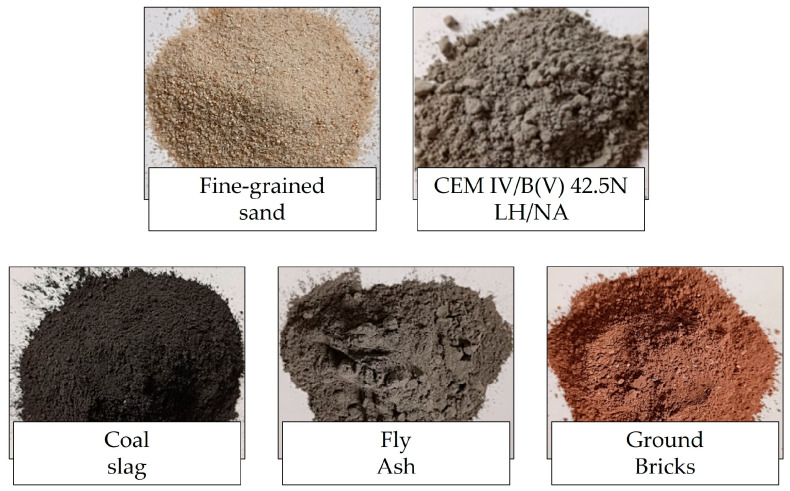
Representative view of raw materials used in the research.

The primary materials utilized in this study included a low-emission cement type CEM IV/B(V) 42.5N LH/NA, supplied by Holcim Group, Małogoszcz Cement Plant (Małogoszcz, Poland); quartz sand; ground construction and demolition waste; fly ash sourced from PGE Energia Ciepła S.A. (Kraków, Poland); and coal slag obtained from Łęczyńska Energetyka Spółka z o.o. (Puchaczów, Poland). The materials used in the experimental program are illustrated in Figure 1. A synthetic foaming agent (PIANOTWÓR, AS, MEEX AG, Chrzanów, Poland) was used to produce the foam. The alkaline activating solution, defined as the medium that dissolves aluminosilicate precursors and initiates geopolymerization, consisted of 10 M sodium hydroxide (NaOH) and sodium silicate (water glass), mixed at a molar ratio of 2.5, yielding a solution with a density of 1.45 g/cm^3^. The water-to-solid (w/s) ratio of the mixtures was maintained in the range of 0.22–0.24. This ratio represents the mass of total water, including that supplied by the alkaline activator, relative to the mass of solid constituents such as aluminosilicate precursors, fillers, and fibers. This parameter governs the rheology, setting behavior, and microstructural development of 3D-printable hybrid geopolymer mixtures or an alkali-activated hybrid binder, thereby determining their printability, mechanical performance, and durability. Tap water was employed for the preparation of the activator solution to closely simulate real-world construction site conditions and to minimize production costs. Quartz sand was intentionally selected as an inert filler due to its non-reactive nature. This choice ensured that any observed changes in material properties could be attributed solely to the tested modifiers rather than to interactions with the aggregate itself. Consequently, the use of quartz sand enhances the experimental reliability and strengthens the scientific validity of the results. Furthermore, the use of a chemically inert and consistent filler improves the reproducibility of the experiments, thereby facilitating accurate verification and replication by other researchers. Reinforcement of the matrix was achieved through the incorporation of both synthetic and natural fibers. In total, six an alkali-activated hybrid binder formulations were prepared, with their detailed compositions summarized in Table 1.

To each composite mixture, pre-generated foam based on the AS synthetic foaming agent was added at a 1:1 volumetric ratio. According to the literature, this ratio represents the upper limit of acceptable foam content, and was therefore selected to evaluate the influence of critical porosity levels on fire resistance. The chosen foam proportion was intended to test the boundary conditions for porosity-induced thermal degradation. Moreover, the fiber dosage was uniformly set at 0.5 wt.% for all series, which corresponds to approximately 0.1–0.4 vol.% depending on fiber density as reported in the authors’ previous study [76]. This level of addition was selected as a compromise ensuring sufficient dispersion and interfacial interaction without agglomeration effects. The fibers employed exhibit aspect ratios in the range of 140–700, which are favorable for crack bridging and stabilization of the pore structure. Higher aspect ratio fibers, such as basalt and glass, are expected to hinder bubble coalescence and drainage, while lower density fibers, such as polypropylene, increase the effective volume fraction at the same weight dosage, thereby contributing to uniform distribution within the matrix and improving shape retention in the context of 3D printability. The selected dosage values were adopted based on previous literature reports and further validated in the authors’ earlier investigations, where they proved effective in maintaining foam stability without negatively affecting the fresh-state rheology.

### 2.2. Mixture Preparation Procedure

**Figure 2:**
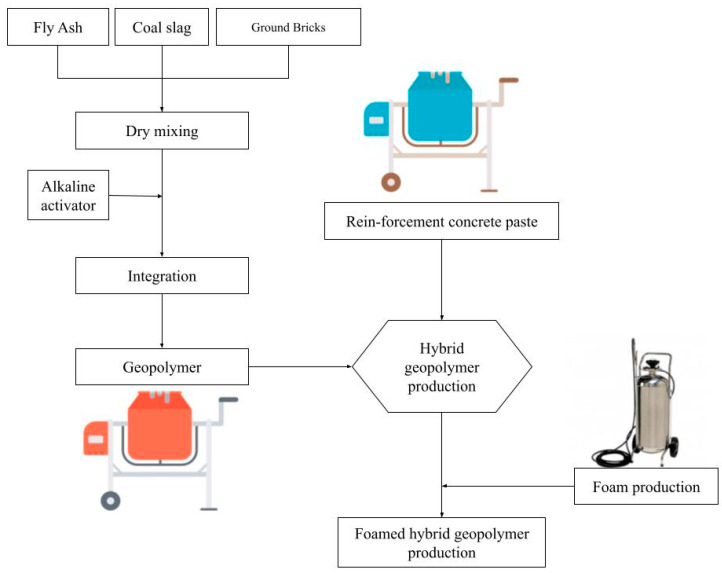
Procedure flow chart of a fiber-reinforced foamed hybrid geopolymer preparation [76,77].

To prepare the geopolymer mortar, coal slag, ground bricks and fly ash were blended for 2 min in a slow-speed industrial mixer (SB-100, Skiva, Płońsk, Poland), powered by a 550 W motor, to ensure thorough homogenization. The alkaline solution was then added, and the mixture was stirred at low speed for 15 min at a rotational speed of 32 revolutions per minute (rpm). Subsequently, the pre-prepared mortar containing dispersed fibers was added and mixed for an additional 2 min. The prefabricated foam was generated using a pneumatic foam generator (BP-4041, BASS POLSKA, Mroków, Poland) with a 25 L capacity. Following the pre-mixing methodology, the foam was then gradually introduced into the fiber-reinforced mortar, and the entire mixture was stirred for a further 2 min to complete the foaming process. The concentration of the foaming agent was maintained at 5%, in accordance with the manufacturer’s recommendations. The mixing speed of 32 rpm was selected to ensure a uniform consistency while preventing the collapse of air voids within the foamed matrix. The foamed pastes were poured into square-plate molds measuring 750 mm × 750 mm with a depth of 150 mm. The primary thickness of the hardened slabs is dependent on foaming effectiveness. Samples were cured under ambient conditions, at a temperature of 22 ± 2 °C and a relative humidity of 55 ± 5%. After four days, the specimens were demolded and continued curing under the same ambient conditions until reaching an age of 28 days. No mechanical compaction (e.g., vibration or tamping) was applied during casting to preserve the internal pore structure created during the foaming process. The schematic representation of the full mixture preparation process, based on data derived from the literature, is provided in Figure 2.

A particular stage in the preparation of the foamed material is the mixing process of the alkali-activated hybrid binder paste with the preformed foam. To ensure the structural integrity of the entrained air voids, the foam must be incorporated gradually into the matrix. This step-wise addition minimizes the risk of bubble rupture or collapse, which could otherwise compromise the homogeneity and porosity of the final composite. The mixing protocol applied in this study was adapted from the procedure previously developed by Markin et al. [78] and is detailed in Table 2.

### 2.3. Methods

#### 2.3.1. Apparent Density

During curing, the specimens were kept at 22 ± 2 °C and 55% relative humidity, conditions chosen to reproduce the environment usually found on construction sites. The test samples were taken from previously prepared slabs, from which uniform prisms measuring 200 × 200 × 50 mm. To determine their apparent density, measurements were carried out with the Lambda HFM 446 thermal conductivity analyzer (Netzsch, Selb, Germany), an instrument that enables precise evaluation of both thermal performance and physical parameters of flat materials.

#### 2.3.2. Small Screening Tests for Fire Resistance

**Figure 3:**
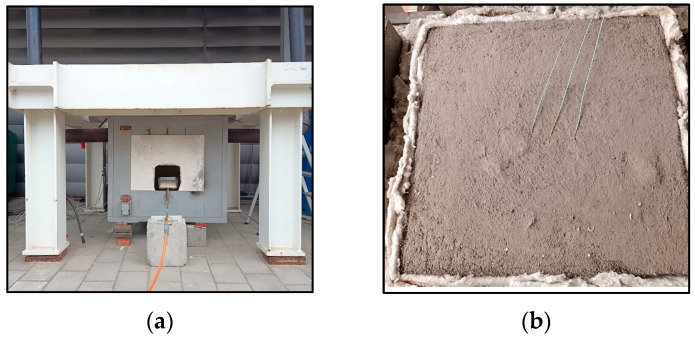
(**a**) View of the furnace used for fire resistance tests, (**b**) sample before fire test with three K-type thermocouples.

The small screening fire resistance tests were conducted using a custom-designed furnace (Dragon, CUT, Kraków, Poland), developed at the Cracow University of Technology [79], as shown in Figure 3a. The apparatus consists of a steel outer shell and an internal chamber lined with rigid refractory ceramic boards and fireclay bricks. The test specimen was inserted into a horizontal opening located in the upper section of the furnace, measuring 600 × 600 mm, which allows for direct fire exposure of the material’s bottom surface. A detailed description of the furnace construction is provided in [80]. For testing purposes, a panel was cut to dimensions of 600 mm × 600 mm × 50 mm and positioned horizontally within the upper chamber of the furnace. Internal furnace temperature was manually regulated using a 140 kW gas burner. The time-temperature scenario followed the standard curve defined by ISO 834-1 for 120 min [80]. On the unexposed (top) side of the specimen, three K-type thermocouples were installed at the mid-length section of the panel to monitor temperature rise and evaluate the thermal insulation performance of the material, as shown in Figure 3b.

During the fire exposure test, the following key observations were recorded:

(a) Monitoring of a self-load-bearing capacity. The self-load-bearing capacity was assessed based on the time the slab could support its own weight before collapsing.
(b) Monitoring of slab integrity. Integrity was evaluated as the duration the slab could prevent flames from passing through to the unexposed side. Integrity was considered lost once flames were detected on the unexposed surface.
(c) Monitoring of thermal insulation. Thermal insulation was determined by measuring the temperature increase on the unexposed surface. Specifically, it was assessed as the time taken for the average temperature (based on three measurements) to rise by 140 °C above the initial value.

To support post-test analysis, photographs of the unexposed surface were taken every 5 min using a digital camera during the fire exposure. In addition, the formation of hot droplets and the release of fumes were also monitored throughout the test. The experimental procedure was intended to provide conditions comparable to the fire resistance evaluation defined in the PN-EN 13501-2 standard [81]. The objective of the fire tests was to compare the fire behavior of the tested materials rather than to determine their fire resistance rating.

#### 2.3.3. Moisture Content

The moisture content of the tested panels was measured prior to fire exposure, immediately after the fire resistance evaluation, and again after a 60-day storage period under standard indoor conditions. Moisture content was determined using a MF 100 Feuchte–Messgerät-type moisture meter (Testo AG, Lenzkirch, Niemcy), with measurements taken at five randomly selected points on the surface of each specimen.

#### 2.3.4. Colorimetry

Surface color changes were examined on samples of the prepared an alkali-activated hybrid binder material. The specimens were cut into cubic dimensions of 50 × 50 × 50 mm and subsequently subjected to controlled thermal treatment in an electric furnace. Heating was performed at a constant rate of 1 °C/min until target temperatures of 200 °C, 400 °C, 600 °C, 800 °C, and 1000 °C were reached. Upon reaching the designated temperature, each specimen was held isothermally for 1 h to ensure uniform temperature distribution throughout the volume. The furnace temperature was monitored using a K-type thermocouple placed in close proximity to the specimen within the furnace chamber. The testing procedure followed established methodologies previously reported in the literature [82,83,84].

Subsequent colorimetric analysis was performed using a high-precision colorimeter (Model NH110, Shenzhen Threenh Technology Co. Ltd., Shenzhen, China). The device employs an 8°/d geometry (8° illumination angle with diffuse reflection detection), which minimizes the influence of surface gloss on measurement accuracy. The light source in the NH110 instrument is an LED emitter with a spectral profile approximating that of illuminant D65, representing standard daylight conditions. Incident light is reflected from the surface of the sample and passed through an optical system equipped with trichromatic filters (R, G, B), simulating the spectral sensitivity of the human visual system. The filtered light is then directed to a silicon photodiode detector, where the electromagnetic radiation is converted into an electrical signal. This signal is processed by an integrated microprocessor, which transforms the data into numerical values in a defined color space. In this study, the CIE Lab color space was employed, as it provides a perceptually uniform representation of color differences and is an extension of the CIE XYZ model. The L* component quantifies lightness (from black to white), while a* and b* represent chromaticity along the green–red and blue–yellow axes, respectively. Prior to measurement, the optical system was calibrated using a standard reference color plate and a defined light source (e.g., D65) to ensure reproducibility and measurement reliability. Even subtle color differences, imperceptible to the human eye, can be detected through numerical analysis of the L*, a*, and b* coordinates. Based on the chromaticity components a* and b*, color saturation (Chroma, C*), which represents the intensity and purity of the color, and hue angle (H*), indicating the tone, were calculated according to Equations (1) and (2):

(1) $$C*=\sqrt{\left({a*}^{2}+{b*}^{2}\right)}$$

where C* is the chroma, *a* represents the red–green axis, and *b* the yellow–blue axis.

(2) $$H*=\tan^{-1}\left(\;\frac{b*}{a*}\right)$$

where H* is the hue angle, *a* and *b* as above. The total color difference (Δ*E*) was calculated according to the Euclidean formula shown in Equation (3):

(3) $$\Delta E=\;\sqrt{{\Delta L*}^{2}+{\Delta a*}^{2}+{∆b*}^{2}}$$

where Δ*E* represents the overall color difference, Δ*L* is the change in lightness, Δa* the change in red–green component, and Δb* the change in yellow–blue component.

Measurements were performed for three distinct layers within each specimen. Layer 1 corresponded to the fire-exposed surface, Layer 2 represented the central section of the cross-section, and Layer 3 was located on the rear face of the panel opposite the flame source where thermocouples had been positioned. For each layer, ten individual measurements were conducted, and mean values were computed to characterize the color parameters of the respective regions, following procedures reported in relevant literature [85,86].

#### 2.3.5. XRF Analysis of Distinct Layer Compositions

The analysis of chemical composition changes in the alkali-activated hybrid binder samples was conducted using X-ray fluorescence (XRF) spectroscopy, employing an EDX-7200 spectrometer (Shimadzu Corporation, Kyoto, Japan).

#### 2.3.6. Computed Tomography Analysis of Porosity

The computed tomography analysis of porosity was carried out using an industrial computed tomography system, Phoenix V|tome|x M240 (Waygate Technologies, Wunstorf, Germany), characterized by a measurement accuracy of (3.8 + L/100 [mm]) µm. The system was equipped with two X-ray tubes: a Microfocus tube (maximum voltage of 240 kV, power of 320 W) and a Nanofocus tube (maximum voltage of 180 kV, power of 20 W). For the purpose of this study, the Nanofocus tube was selected due to its superior spatial resolution of 0.2 µm. Data acquisition was performed using the Datosx 2 acq software, which controls the CT system. The scan parameters were set as follows: voltage 160 kV, current 220 µA, sensitivity 1, projection time 100 ms, number of projections 2000, and ambient temperature during measurement 20 °C.

#### 2.3.7. Microscopy Analysis

The surface topography of the foamed materials was observed using a digital optical microscope (Keyence VHX-7000, KEYENCE International, Mechelen, Belgium). The analysis was conducted in multi-plane observation mode, enabling the acquisition of high-resolution images with extended depth of field. Sample surfaces were observed at various magnifications, allowing for a detailed assessment of pore morphology, pore distribution, and structural defects. Image processing and quantitative analysis were carried out using Fiji ImageJ software (version IJ 1.46r).

## 3. Results and Discussion

### 3.1. Volumetric and Moisture Changes During Curing

**Figure 4:**
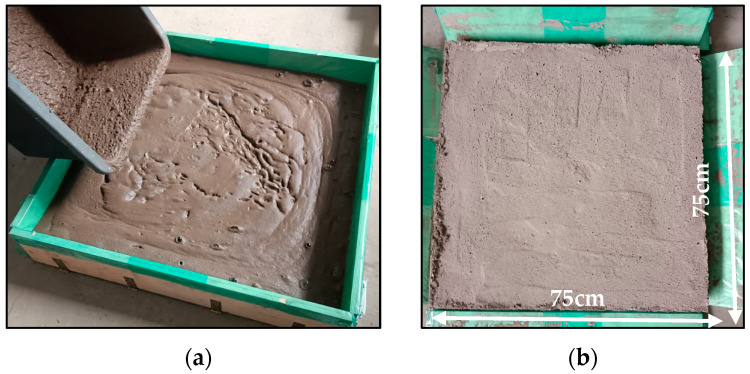
Representative view of the samples: (**a**) as-cast, (**b**) at the stage of demolding.

**Figure 5:**
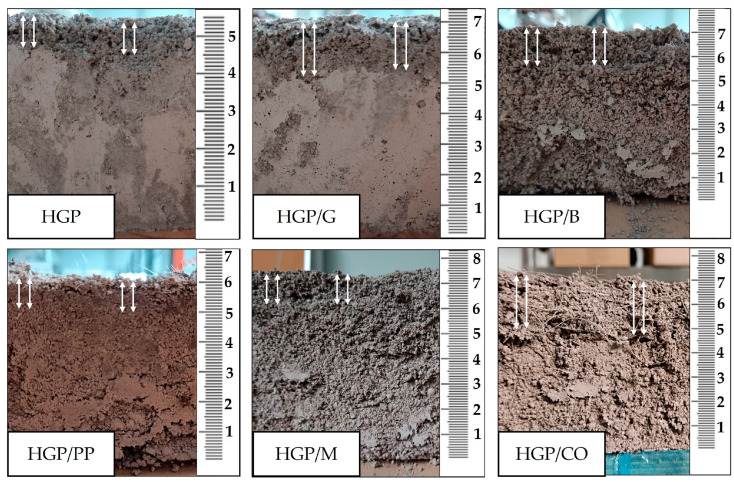
Representative cross-section of cast samples, with foam regions indicated by arrows; Image recorded after 28-day curing period.

Following the casting procedure, surface leveling was performed, and the vertical dimension of each specimen was measured at five equidistant points per material formulation to assess casting uniformity and dimensional consistency (Figure 4). After 28 days of ambient curing, post-curing height measurements were conducted to quantify foam settlement and assess structural stability over time. The measurement data are summarized in Table 2. Visual inspection upon demolding revealed distinct phase separation in all specimens, characterized by the accumulation of foam in the upper zones of the cast bodies. This indicates gravitational segregation during the setting process. This stratification resulted in a mechanically compromised, brittle top layer with reduced structural integrity, as documented in Figure 5. The height shown in Figure 5 corresponds to the lateral face of the demolded specimens, and the photographs were taken after 28 days of curing. The smoother surfaces observed for the fiber-free and glass fiber-reinforced samples result from their higher apparent density and more compact microstructure, whereas the remaining mixtures exhibited an extensive network of open pores throughout the entire volume. The observed phase separation and accumulation of foam in the upper region of the specimens may be attributed to the behavior of the fresh mixtures. Possible contributing factors include relatively low viscosity of the slurry or slower setting kinetics, which could facilitate upward migration of entrapped air and formation of a porous top layer. Such stratification highlights the sensitivity of the system to mixture composition and fresh-state properties, as it reduces structural uniformity between the lower and upper zones of the specimens, introduces local gradients in porosity and density, and ultimately affects the reproducibility of material performance. In the context of 3D printing, such stratification may also influence the thermal insulation performance of the material, as the porous top layers formed by foam accumulation could locally enhance insulating capacity, but at the expense of structural uniformity and repeatability of printed elements. The specimen heights used for fire resistance testing correspond to the values listed in Table 3 under average of measurements taken after 28 days and were subsequently reduced to 5 cm to meet the technical requirements of the furnace, with the cut made on the porous side. To mitigate geometric variability and ensure reproducibility in subsequent experimental procedures, all specimens were precision-ground to a uniform height of 50 mm, thereby eliminating surface irregularities and standardizing sample dimensions for comparative analysis.

**Figure 6:**
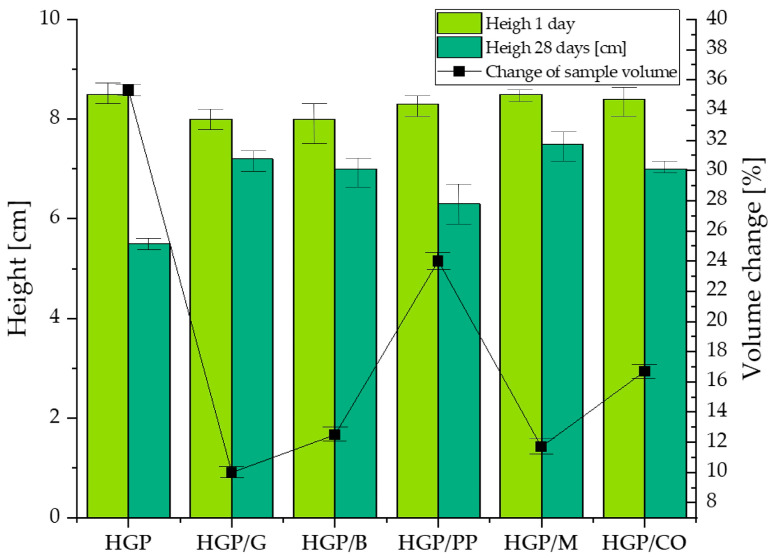
Average of sample heights as a function of mixture composition and curing time, with corresponding sample volume change after 28 days of curing.

Figure 6 presents the initial thickness of the specimens as a function of mixture composition and curing time, along with the corresponding volume change after 28 days. The initial specimen height was subsequently reduced to 5 cm for the fire resistance tests, in compliance with the technical requirements of the furnace. The trimming was performed on the porous side of the specimens, as this surface contained the loosely bonded material.

**Figure 7:**
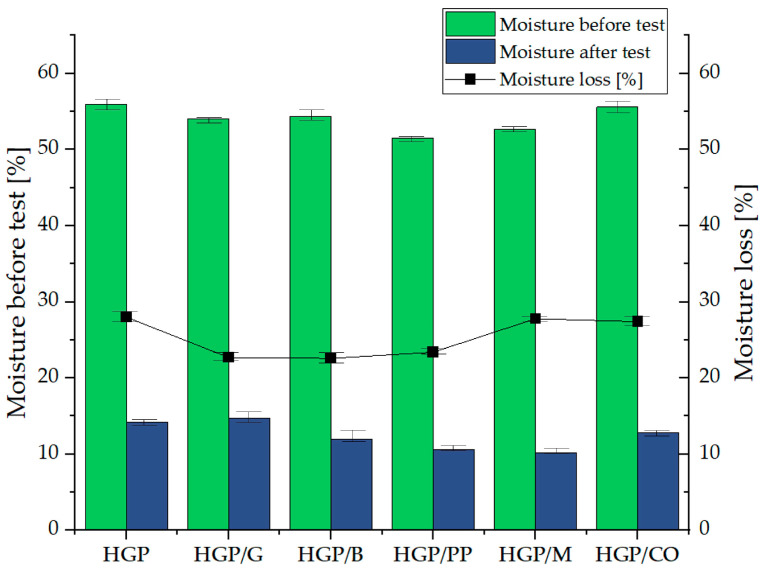
Moisture of samples as a function of mixture composition and fire resistance testing, and after 60 days after fire testing in atmospheric conditions.

An evaluation of the prepared composite samples in terms of volumetric stability (Figure 6) and moisture content variation (Figure 7) revealed distinct correlations between these parameters. The fiber-free specimen (HGP) with the highest density (1053.66 kg/m^3^), exhibited the most pronounced volumetric contraction over the 28-day curing period, amounting to 35.3%. This sample also experienced a substantial reduction in moisture content following the post-fire-exposure condition, decreasing from 56.0% to 14.2%. These observations suggest a high susceptibility of the HGP matrix to shrinkage and moisture loss, likely attributable to its inherently porous structure and elevated initial water content. In contrast, the HGP/PP sample, characterized by a lower density (742.30 kg/m^3^), demonstrated a less severe volumetric decrease (24.0%) and a similarly notable reduction in moisture content (from 51.5% to 10.57%), indicating comparable mechanisms of dehydration and shrinkage, albeit of reduced intensity. The sample reinforced with glass fibers (HGP/G), with a density of 753.60 kg/m^3^, exhibited the lowest volumetric shrinkage among all tested compositions (10.0%), suggesting enhanced structural stability during curing. Nevertheless, this specimen displayed the highest moisture loss post-exposure, with a decline from 60.0% to 14.75%, likely due to its high initial water absorption capacity and subsequent rapid release under elevated thermal conditions. Samples HGP/CO and HGP/B having densities of 742.75 and 741.96 kg/m^3^, respectively, presented moderate volumetric reductions (16.7% and 12.5%, respectively), accompanied by significant moisture decreases (to 12.82% and 12.01%, respectively), indicating relatively stable matrices that are nonetheless prone to dehydration under high-temperature exposure. The HGP/M specimen, with a density of 742.47 kg/m^3^ despite its initially greater dimensions (85 mm), exhibited moderate reductions in both volume (11.7%) and moisture content (from 52.7% to 10.21%), reflecting a comparatively stable microstructure with limited susceptibility to thermal shrinkage and moisture release.

### 3.2. Small Screening Tests for Fire Resistance

**Figure 8:**
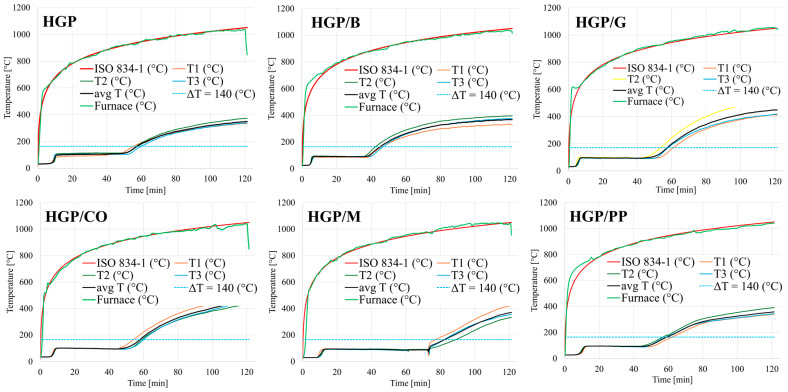
The measurements from fire tests for each sample: the scatter of measurements for temperature rise on the unheated surface (T1, T2, T3), average temperature from 3 measurements (avg T), standard curve ISO 834-1 and development of temperature in the furnace measured by plate thermocouple (Furnace).

**Figure 9:**
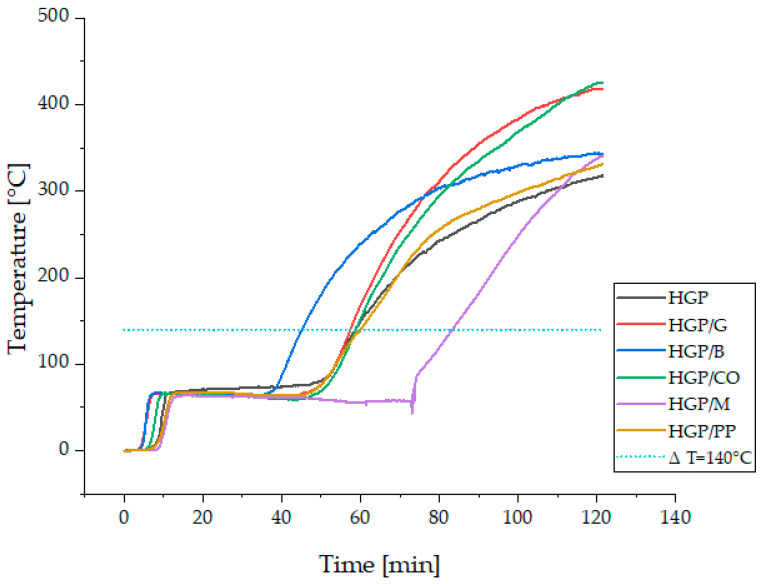
Comparison of the temperature rise on the unheated surface for tested materials(average of three measurements).

**Figure 10:**
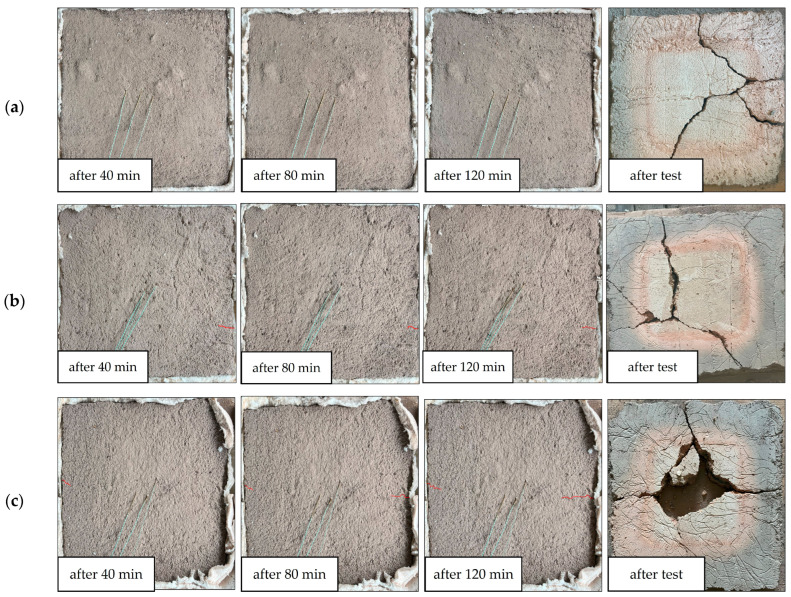

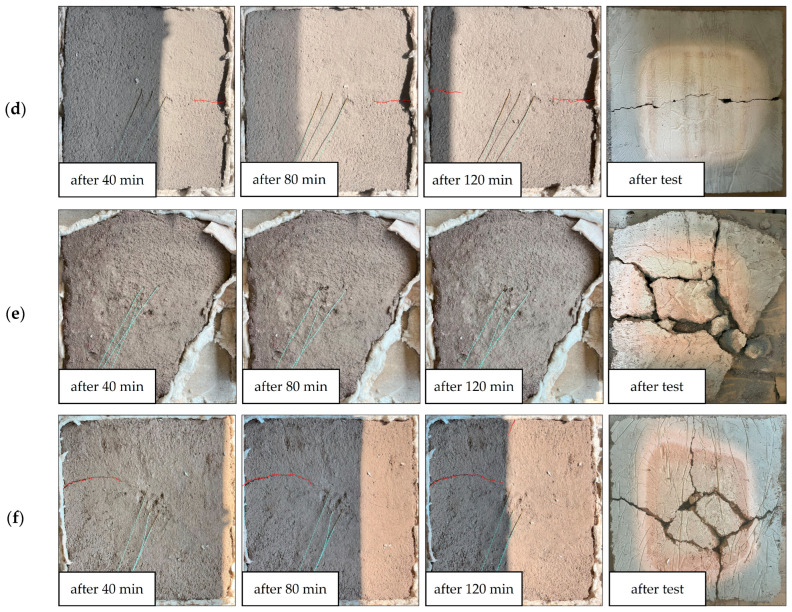
Representative view of the unexposed (top) side of the samples 60 × 60 cm: (**a**) HGP, (**b**) HGP/G, (**c**) HGP/B, (**d**) HGP/PP, (**e**) HGP/M, and (**f**) HGP/CO, depending on the fire exposure time. Cracks observed on the plate surfaces were highlighted in red.

During the fire resistance test conducted using the furnace (Figure 3), panel integrity was assessed in terms of the material’s capacity to prevent flame breakthrough on the fire-exposed surface over a specified time interval. Simultaneously, real-time temperature monitoring was performed on the unexposed surface to quantify thermal insulation performance, as shown in Figure 8. The insulation failure criterion was established as an increase of 140 °C in the average surface temperature relative to the initial baseline, in accordance with standardized fire resistance testing methodologies. A comparative analysis of the temperature rise on the unheated surfaces of the tested materials (averaged over three measurements) further illustrates these trends (Figure 9). To facilitate comprehensive analysis, high-resolution photographic documentation of the unexposed surface was captured at 5 min intervals using a digital imaging system, enabling visual tracking of material degradation, cracking, and thermal effects throughout the exposure period (Figure 10).

The results of the temperature rise on the unexposed surface recorded during the post-fire-exposure condition are presented in the graph shown in Figure 8 and Figure 9. For the comparison in Figure 9, the initial temperature at the fire initiation was set to 0 °C to enable clear assessment of thermal insulation properties between tested formulations. The real initial temperature ranged between 22 and 26 °C.

Among all the tested samples, it is clearly noticed that the composite containing merino wool fibers (HGP/M) exhibited the most favorable fire insulation performance. The time required for the temperature on the unexposed surface to increase by 140 °C, the defined threshold for loss of fire insulation, was recorded at 83 min, representing the longest duration among all tested specimens. The temperature profile for this sample (Figure 8 and Figure 9) clearly demonstrates a delayed temperature rise, confirming the effectiveness of merino wool as a thermal insulating additive. This performance may be attributed to the high air retention capacity and low thermal conductivity of the wool fibers. Conversely, the sample reinforced with basalt fibers (HGP/B) exhibited the lowest fire insulation efficiency, with the 140 °C threshold reached after only 45 min. The temperature increase in this case was notably rapid, suggesting high thermal conductivity of the fibers and limited capacity to impede heat transfer. Although basalt fibers are inherently resistant to high temperatures, their rigidity and limited capacity to dissipate thermal stress may have contributed to early degradation of the composite structure [87,88]. The alkali-activated hybrid binder composite reinforced with synthetic polypropylene fibers (HGP/PP) demonstrated moderate insulation performance, maintaining its insulating capacity for 60 min. For the majority of the testing period, the specimen effectively resisted temperature rise; however, an accelerated increase was observed between 50 and 70 min, corresponding with the melting point of polypropylene (160–170 °C). Fiber melting likely reduced the structural integrity of the matrix, resulting in increased thermal permeability. In the sample containing plant-based coconut fibers (HGP/CO), the fire insulation threshold was maintained for 58 min. However, a visible surface crack on the unexposed face emerged after approximately 40 min and progressively deepened during exposure (Figure 10f). This observation indicates that the material underwent structural degradation under thermal stress, and that the fibers were unable to maintain matrix continuity, likely due to the limited thermal stability of the organic reinforcement. Although coconut fibers possess moderate fire resistance, their degradation compromised the structural reinforcement of the panel, possibly due to their organic nature and susceptibility to prolonged heat exposure. The sample containing glass fibers (HGP/G) achieved a fire insulation duration of 57 min. The temperature profile was approximately linear throughout the test, although a slight acceleration was observed after 40 min. Despite the high melting point of glass, limited adhesion to the alkali-activated hybrid binder matrix and susceptibility to chemical corrosion may have diminished the long-term effectiveness of the reinforcement. For comparison, the unreinforced reference sample (HGP) maintained fire insulation for 58 min, placing it in the mid-range of all results. Despite the absence of fiber reinforcement, no visible cracking or material failure was observed, even after the full 120 min test duration. This suggests that a dense, compact alkali-activated hybrid binder matrix without reinforcement can, under certain conditions, provide sufficient resistance to high-temperature exposure. While the lack of fibers inherently eliminates crack-bridging mechanisms, no evidence of such failure modes was observed, indicating the thermal stability of the unreinforced matrix within the parameters of this study. At the same time, recent advances in additive manufacturing (3D printing) offer promising approaches to further improve the performance of alkali-activated hybrid binders, particularly in addressing challenges such as volumetric instability, curing-induced shrinkage, and fire resistance. For instance, Ziejewska et al. [89] demonstrated that 3D-printed concrete–geopolymer hybrids retained superior residual compressive strength after fire exposure compared to conventionally cast samples. Taken together, these observations suggest that 3D printing can enhance both volumetric and thermal stability in geopolymers, thereby complementing and extending the insights obtained in the present work.

### 3.3. Colorimetry

**Figure 11:**
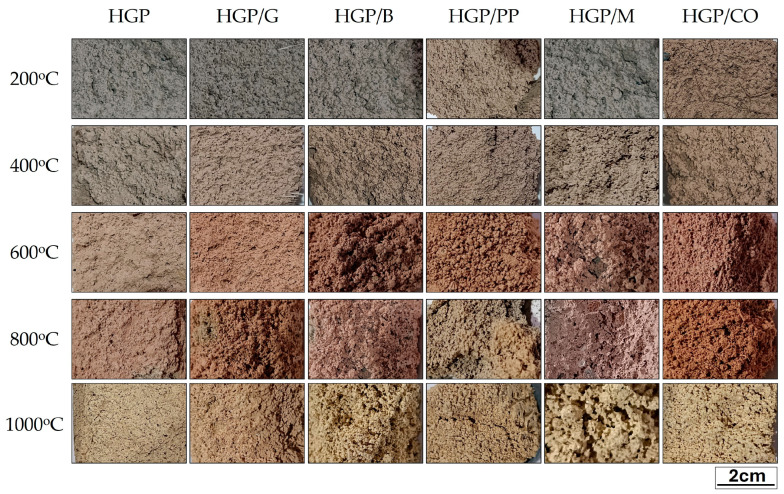
Color change in fiber-reinforced alkali-activated hybrid binder samples depending on the mixture composition and the annealing temperature.

Colorimetric analysis is a valuable diagnostic tool for assessing thermally induced damage in cementitious materials. It enables estimation of the maximum temperature reached by quantifying surface color changes, which often reveal internal damage not visible to the eye. Literature shows that concrete exhibits characteristic color shifts with rising temperature: reddish hues at 300–600 °C, whitish-gray tones at 600–900 °C, and yellowish-ochre shades above 900 °C. In this study, color variations were examined on fiber-reinforced foamed alkali-activated hybrid binder exposed for one hour at 200–1000 °C [82,83,90,91,92]. Figure 11 presents photographs of representative, non-fire-tested samples that were subjected to controlled thermal exposure for one hour at the target temperatures. These specimens were subsequently analyzed using a precision colorimeter to quantify color variation in terms of ΔE values (Table 3). Image-based observation revealed that moderate color changes were already apparent at 200 °C; notably, samples HGP/CO and HGP/PP exhibited early signs of discoloration even at these relatively low temperatures. At 600 °C, all samples exhibited a significant increase in ΔE, indicating pronounced thermal degradation and the development of reddish hues, in agreement with previous findings. The exception was the HGP/M sample (ΔE = 5.38), which showed comparatively minor color deviation, suggesting superior thermal stability or reduced heat penetration. Further heating to 800 °C resulted in a paradoxical decrease in ΔE values, despite the ongoing progression of high-temperature transformations; at this stage, ΔE ranged from 6.05 to 12.76. At 1000 °C, color change levels appeared to stabilize, with ΔE values between 8.07 and 15.39 across all samples. Although this apparent “stabilization” may suggest visual uniformity, underlying mineralogical changes were profound. Yellow and ochre tones became dominant, indicative of advanced chemical decomposition and the formation of new high-temperature crystalline phases such as mullite and nepheline.

**Figure 12:**
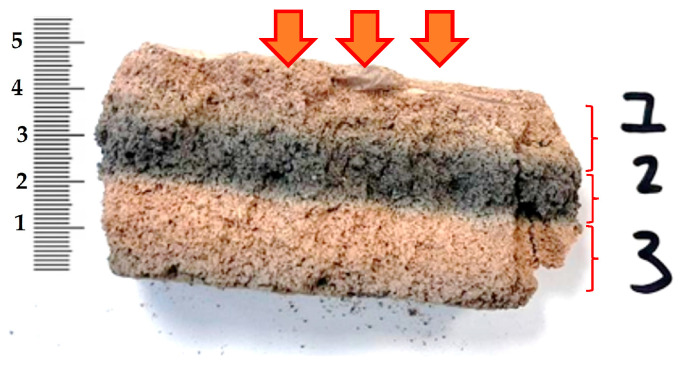
Representative view of cross-section of the samples following fire-exposure testing, with the examined layers identified. Layer 1—fire-exposed surface, indicated with arrows; Layer 2—central region of the cross-section; Layer 3—rear face of the panel opposite the flame source, where thermocouples were positioned.

Subsequent to the simulated fire conditions, advanced colorimetric analyses were performed using the NH110 high-precision colorimeter to quantitatively evaluate thermally induced chromatic alterations across the depth of the exposed alkali-activated hybrid binder specimens. As detailed in Table 4 and illustrated in Figure 12, color difference values (ΔE) were determined for three zones: the fire-exposed surface (Layer 1), the internal cross-section (Layer 2), and the rear, non-exposed surface (Layer 3). In all cases, notable interlayer ΔE variations were observed, confirming that thermal exposure exerts a substantial effect on both surface appearance and subsurface composition. For the HGP/CO specimen, ΔE values reached 4.82 (Layer 1), peaked at 19.69 (Layer 2), and remained perceptible at 7.63 (Layer 3), indicating significant thermal alteration even in zones shielded from direct flame contact. The basalt fiber-reinforced composite (HGP/B) exhibited ΔE values exceeding 11 across all layers, denoting a complete chromatic transformation throughout the material’s thickness. Similar profiles were recorded for HGP/G and HGP/PP, with ΔE in Layer 1 surpassing 12, and moderate but still substantial discoloration persisting in Layer 3. The HGP/M composite demonstrated a distinct chromatic gradient: ΔE values of 7.50 (Layer 1), 11.42 (Layer 2), and 3.97 (Layer 3) implying attenuated thermal diffusion and enhanced thermal shielding associated with the wool-fiber matrix. Remarkably, even the unreinforced reference specimen (HGP) exhibited ΔE values of 11.49, 7.61, and 6.31 in Layers 1, 2, and 3, respectively, affirming that the alkali-activated hybrid binder matrix itself undergoes measurable colorimetric transformation under elevated thermal loads.

**Figure 13:**
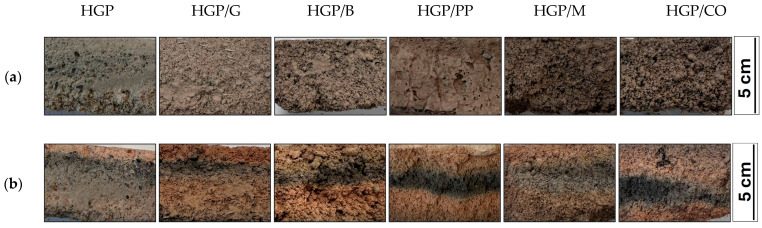
Representative view of samples cross-section: (**a**) before simulated fire conditions, (**b**) after fire-resistance evaluation.

The majority of the recorded color difference (ΔE) values exceeded the established perceptibility threshold of 3.5, with several values surpassing 10, thereby indicating perceptible to pronounced chromatic deviations between the pre- and post-exposure states of the samples. These results underscore the potential of colorimetric analysis as a non-destructive diagnostic technique for estimating the extent of thermal penetration and for characterizing heat-induced deterioration in alkali-activated hybrid binder composites. Exposure to elevated temperatures is known to initiate mineralogical transformations such as the crystallization of high-temperature phases, including mullite and nepheline, which frequently manifest as measurable alterations in surface coloration [93]. Colorimetric evaluation thus offers a quantitative means of assessing thermally induced changes not only on the exposed surface but also within sub-surface strata of alkali-activated hybrid binder matrices. This methodology holds promise as a supplementary quality assurance tool in the manufacturing of geopolymer and alkali-activated hybrid binder composites designed for high-temperature environments. A visual comparison is provided in Figure 13, depicting cross-sections of an alkali-activated hybrid binder specimens prior to (a) and subsequent to (b) standardized fire-exposure simulation. This visual documentation supports further interpretation of the relationship between thermally induced mineralogical changes and the corresponding colorimetric signatures, thereby enhancing the material diagnostic framework.

While previous studies on 3D-printable geopolymers and geopolymer foams have primarily concentrated on rheological behavior, microstructural development, and thermal insulation properties, they have not incorporated colorimetric methods to evaluate thermal resistance. The results of the present study address this gap by integrating classical diagnostic techniques, such as colorimetric analysis and ΔE quantification, with the characterization of materials that can feasibly be adapted for process control in 3D printing. In the next stage of this research, the developed mixtures will be directly employed in 3D printing to verify their performance under layer-by-layer deposition and to evaluate their durability in high-temperature environments. Incorporating these insights, the colorimetric findings presented herein not only establish a robust diagnostic framework for thermally induced degradation but also reinforce the potential of alkali-activated hybrid binder foams as viable candidate materials for additive manufacturing applications in high-temperature scenarios.

### 3.4. XRF Analysis of Distinct Layer Compositions

**Figure 14:**
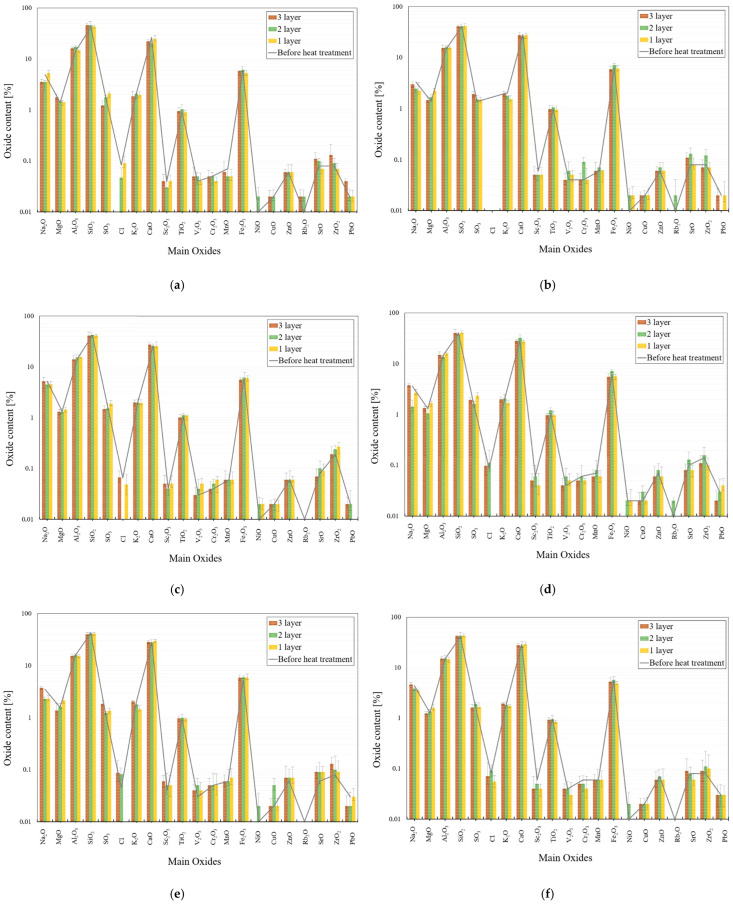
XRF analysis of samples (**a**) HGP/CO, (**b**) HGP/B, (**c**) HGP/G, (**d**) HGP/M, (**e**) HGP/PP, and (**f**) HGP after the fire resistance test, presented as a function of the identified layers: Layer 1—fire-exposed surface; Layer 2—central region of the cross-section; Layer 3—rear face of the panel opposite the flame source, where thermocouples were positioned.

The X-ray fluorescence (XRF) analysis (Figure 14) was conducted on a reference specimen and on each of the three compositional layers exposed after the fire resistance test, as defined in Figure 12. Although the overall composition remains constant, reinforcing fibers influence material behavior under fire exposure. The evaluation highlights oxide-specific compositional variations with depth, emphasizing their spatial distribution across the layers (Figure 14). Sodium oxide (Na_2_O) exhibits pronounced spatial and thermal mobility, particularly within Layer 1, which is directly subjected to flame impingement. Here, elevated thermal gradients facilitate significant ionic diffusion and partial volatilization of Na_2_O. Notably, its concentration displays bidirectional trends, with increases observed in certain composite systems (e.g., HGP/CO) and concomitant reductions in others an outcome that reflects material-dependent volatility thresholds and microstructural pathways for mass transport. In the outermost Layer 3, Na_2_O content is consistently and markedly depleted, attributable to its high volatility and subsequent sublimation into the ambient atmosphere [94,95]. Fibrous phase properties slightly affect sodium migration, but the main transport mechanism remains unchanged. Magnesium oxide (MgO), owing to its high thermodynamic stability under oxidative and high-temperature regimes, exhibits a relative enrichment in Layers 1 and 2. This enrichment is primarily attributed to the volatilization and depletion of more labile oxides such as Na_2_O and K_2_O, resulting in a concentration-driven increase in MgO content. Fiber-related heterogeneities in heat conduction and localized microstructural anisotropy may further influence Mg redistribution at the microscale, although these effects remain secondary. Aluminum oxide (Al_2_O_3_), the most thermally stable of the oxides analyzed, accumulates predominantly within Layer 2. This intermediate zone effectively acts as a thermally stabilized diffusion barrier, in which Al_2_O_3_ consolidates into a refractory framework that retards further decomposition and structural destabilization upon fire exposure. In the case of HGP/CO, a pronounced enrichment of silicon dioxide (SiO_2_) is observed in Layers 2 and 3, indicating preferential retention and post-volatilization concentration of silica following the depletion of less stable oxides. While other composites demonstrate less pronounced variations in SiO_2_, a general trend of increased concentrations in these layers relative to the reference specimen is evident, most plausibly associated with the in situ formation of thermally stable silicate glass phases that enhance both mechanical and thermal stability [96,97]. Sulfur trioxide (SO_3_) demonstrates a localized increase in Layer 1, particularly in HGP/CO (from 1.7% to 2.1%) and HGP/M (from 1.54% to 2.37%). This enrichment is hypothesized to result from the pyrolytic degradation of organic constituents and/or secondary reactions with sulfur-containing volatiles released during fiber combustion [98,99] n contrast, Layer 3 typically exhibits SO_3_ concentrations that are either commensurate with or slightly lower than those of the reference sample. Chlorine (Cl), present at low baseline concentrations, exhibits behavior governed predominantly by its high vapor pressure and chemical reactivity. Volatilization leads to its progressive depletion in Layer 3, whereas partial retention and localized accumulation may occur within microfractures in Layer 1, where rapid thermal cycling may inhibit its full volatilization. Potassium oxide (K_2_O) undergoes significant depletion within Layer 1, which can be ascribed to the low boiling point of potassium and the facile formation of volatile potassium-containing compounds under fire conditions [100]. Calcium oxide (CaO) concentrations in Layer 1 remain essentially stable, with only marginal variations, indicating resilience under thermal loading. Minor oxides such as TiO_2_, V_2_O_5_, MnO, Cr_2_O_3_, and ZnO show negligible, non-systematic fluctuations, reflecting thermal inertness and limited mobility; this is particularly evident in HGP/CO, where compositional profiles closely resemble those of the unexposed reference. Fiber typology mainly influences mesoscale microstructure and anisotropic distributions of heat flux and stress, thereby indirectly modulating oxide migration and phase behavior. Nevertheless, the dominant transformation pathways, including volatilization of fugitive species, stabilization through refractory oxide formation, and Ca–S compound interactions, remain consistent across the system. Collectively, these processes drive a distinct, layer-specific redistribution of oxides within the matrix under flame exposure near 1000 °C.

### 3.5. Computed Tomography Analysis of Porosity

**Figure 15:**
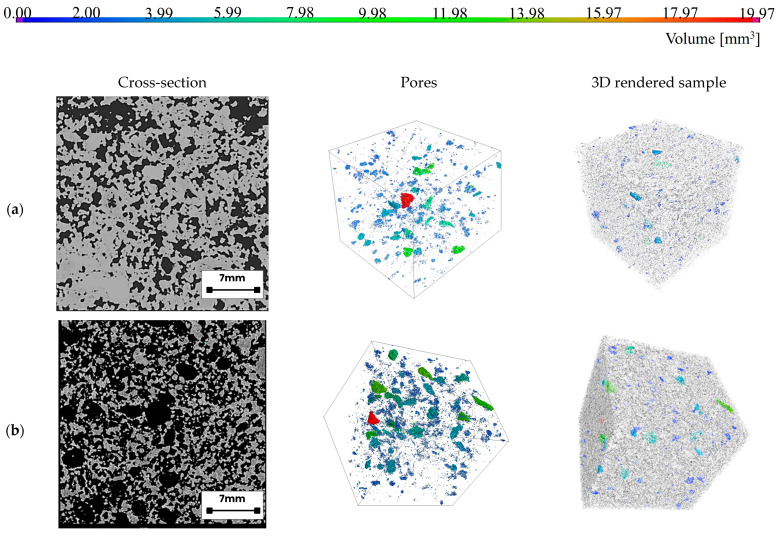

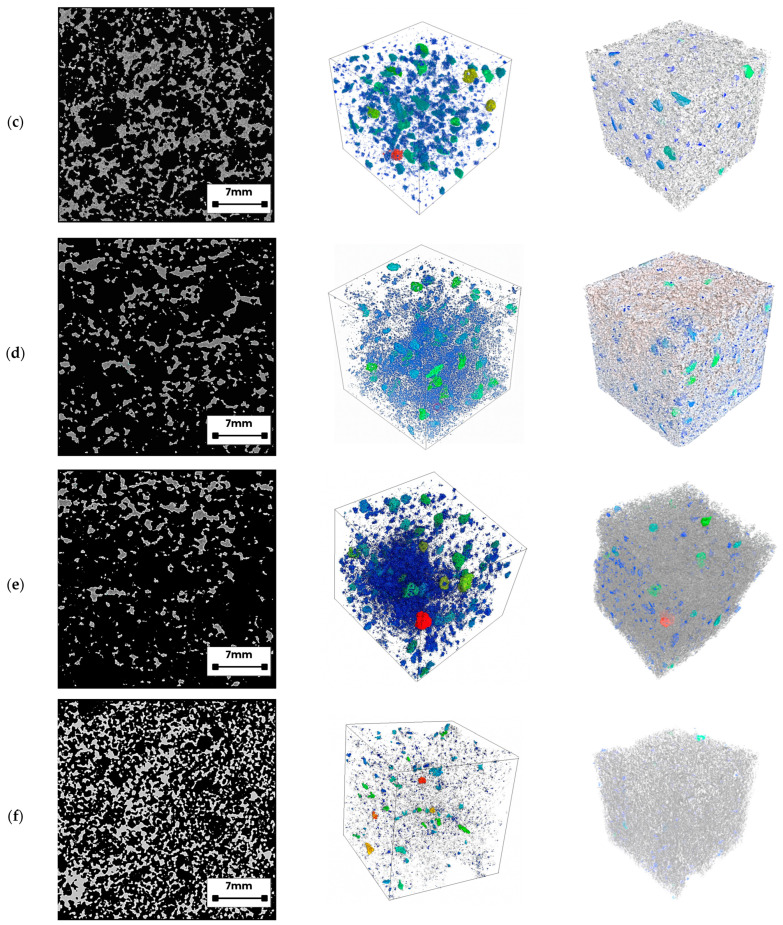
Cross-section and 3D visualization of porosity obtained from CT analysis, and the 3D render of samples: (**a**) HGP, (**b**) HGP/G, (**c**) HGP/B, (**d**) HGP/PP, (**e**) HGP/M, and (**f**) HGP/CO.

X-ray computed tomography (XCT) was employed for the three-dimensional visualization and spatial characterization of the pore architecture in fiber-reinforced alkali-activated hybrid binder foams. The samples analyzed, as listed in Table 5, encompassed a region approximately 40 × 40 mm in size. The reconstructed tomographic images presented in Figure 15 substantiate the quantitative porosity data obtained for the studied alkali-activated hybrid binders. The reference sample, HGP (a), exhibited an almost entirely closed and compact microstructure with a minimal pore population, consistent with its lowest measured relative porosity (11.79%) and the highest fraction of closed porosity (6.29%). In the 3D rendering, pores appear sparsely distributed and poorly interconnected, confirming their dense, near-impermeable morphology. In contrast, the HGP/G (b) specimen displayed a substantial increase in both pore number and total pore volume. The microstructure transitioned toward a more open-cell configuration, as indicated by a high relative porosity of 41.44% and a minimal closed porosity of 0.18%. The 3D model reveals large, interconnected voids of variable size, reflecting the emergence of a more developed porous network. HGP/B (c) demonstrated an even more pronounced porous structure, characterized by a high density of voluminous, spatially continuous pores, consistent with its highest recorded total pore volume (19,191 mm^3^) and relative porosity (55.19%), alongside a nearly negligible closed porosity fraction (0.01%). Despite this, the sample exhibited the poorest thermal insulation performance, suggesting that large, coalesced pores may facilitate rather than hinder heat transfer, indicating that not only pore quantity but also their size distribution, spatial arrangement, and interconnectivity are critical parameters governing thermal insulation efficiency [101,102]. This suggests that not merely the total pore volume, but more critically the pore size, spatial distribution, and degree of interconnectivity play a pivotal role in governing the fire-resistant properties of the material [103]. The HGP/PP (d) sample exhibited an exceptionally high relative porosity (66.86%) and considerable total pore volume (14,191 mm^3^). The cross-sectional image shows a dense population of pores, while the 3D reconstruction highlights their irregular morphologies and branched networks. The closed porosity remained very low (0.01%), indicating the dominance of open-cell structures. HGP/PP exhibited moderate thermal resistance, implying that while the high porosity may delay heat transfer, the large and irregularly interconnected pore network may compromise the overall thermal barrier performance. Although increased air voids theoretically lower thermal conductivity, excessively large or percolated pores may facilitate heat penetration rather than inhibit it. The HGP/M (e) specimen demonstrated the most extensively developed porous network, with the highest measured relative porosity (74.37%) despite a comparatively moderate total pore volume (10,989 mm^3^). Cross-sectional analysis revealed a high concentration of fine pores, and the 3D rendering displayed a compact yet highly foamed structure composed predominantly of interconnected microvoids. Such a configuration is conducive to effective thermal energy dissipation and absorption, thereby enhancing thermal insulation efficiency. Similar observations have been reported in the literature. Novais et al. [104] presented a systematic overview of geopolymer foams with controlled porosity, emphasizing their potential for energy-efficient construction and their compatibility with additive manufacturing approaches. This configuration—dominated by small, tightly packed, and interconnected poresis conducive to effective thermal energy dissipation and absorption, thereby reducing thermal conductivity. Finally, the HGP/CO (f) sample also displayed an advanced porous structure, with a relative porosity of 61.77% and a total pore volume of 16,378 mm^3^. Both the cross-sectional imaging and the 3D visualization reveal a diverse array of pore sizes, including prominent large voids. The low closed porosity value (0.36%) confirms a microstructure composed primarily of open pores with isolated closed cavities. These results are in agreement with the porosity metrics summarized in Table 5, highlighting marked differences between the dense reference sample (HGP) and the fiber-modified hybrid systems. The progressive incorporation of various additives (G, B, PP, M, CO) transforms the material structure from a compact, low-porosity matrix into an increasingly open and morphologically complex porous architecture. This structural evolution significantly influences not only the material’s thermal transport properties but also its mechanical behavior and functional performance under high-temperature conditions.

In this context, recent advances in additive manufacturing (3D printing) have shown that alkali-activated and geopolymer foams can be fabricated with tailored pore structures, which strongly determine their thermal and mechanical performance. For example, Alghamdi et al. [105] demonstrated the extrusion-based printing of fly ash geopolymer foams with controlled porosity and thermal insulation potential. Barve et al. [106] emphasized the influence of rheology and printing parameters on pore distribution and microstructural anisotropy in 3D-printed geopolymers. Other research highlighted the potential of foam 3D printing for lightweight and insulating building elements, demonstrating how printing patterns affect heat transfer and compressive strength [107].

The results obtained in the present study confirm the potential of the developed alkali-activated hybrid binder foams for 3D printing applications. In this form, the mixture can be further adapted for extrusion-based additive manufacturing, which will be the subject of the authors’ future research.

### 3.6. Optical Microscopy Analysis

**Figure 16:**
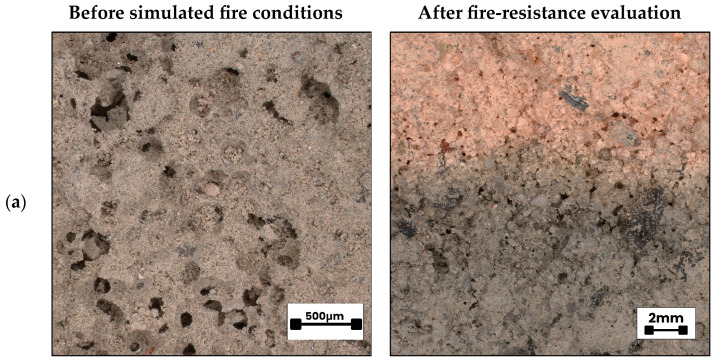

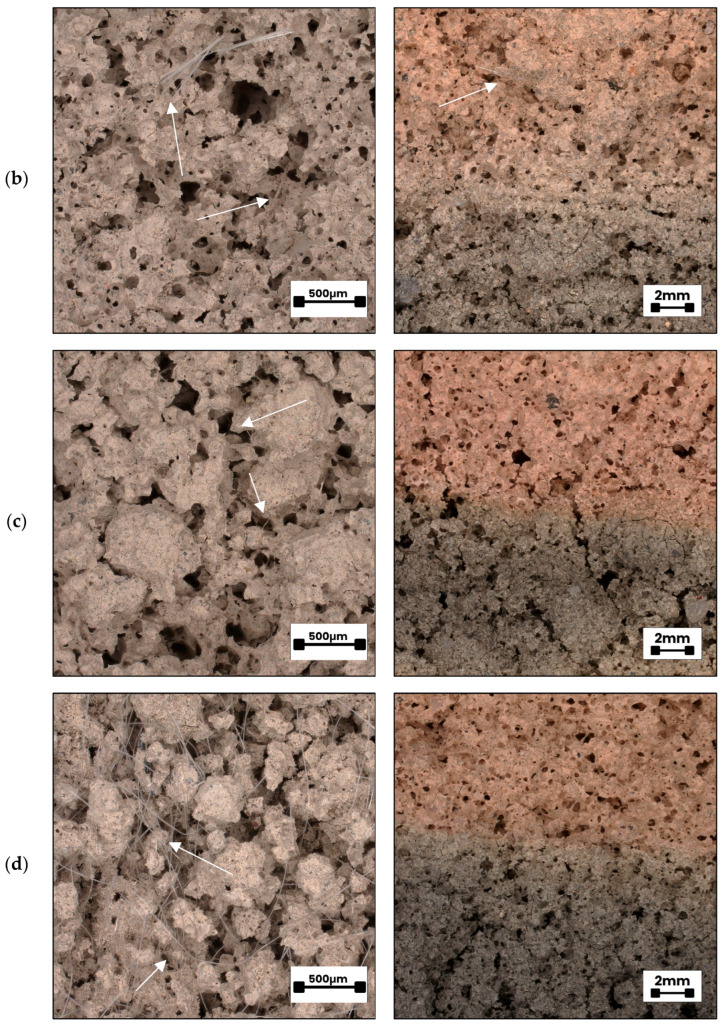

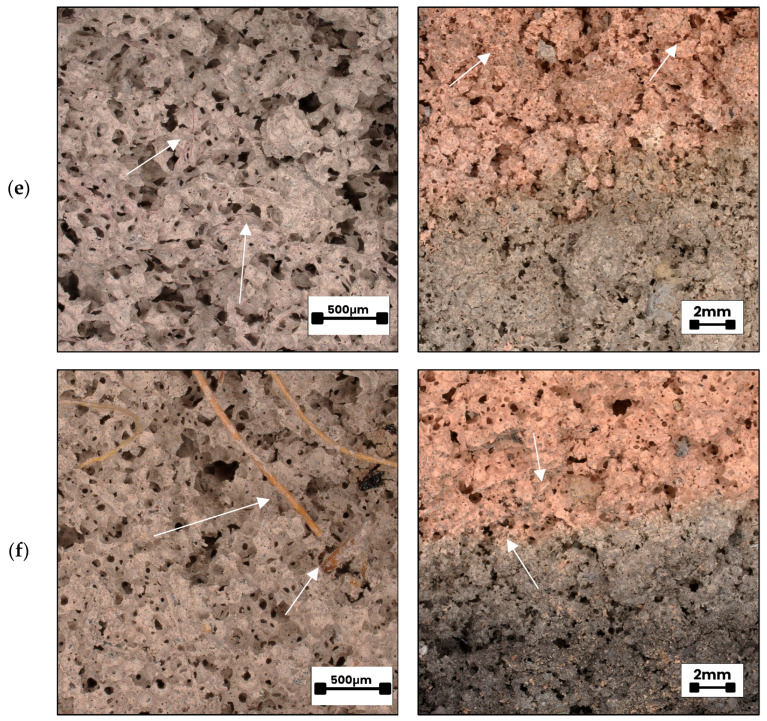
Microstructural comparison of pore morphology, fiber distribution, and fiber-matrix interface integrity before simulated fire exposure (**left**) and after fire-resistance evaluation (**right**) for samples: (**a**) HGP, (**b**) HGP/G, (**c**) HGP/B, (**d**) HGP/PP, (**e**) HGP/M, and (**f**) HGP/CO. Arrows indicate examples of fiber locations.

Figure 16 presents the microstructural characteristics of previously examined foamed alkali-activated hybrid binder specimens, documented prior to simulated fire exposure (left column) and after fire-resistance evaluation (right column). Images were acquired using a digital optical microscope at two magnification levels (500 µm and 2 mm scales) to facilitate a detailed assessment of the porous matrix, fiber distribution, and fiber–matrix interfacial bonding both before and after thermal loading. Across all compositions, the left-hand micrographs (pre-exposure) reveal the initial pore morphology and the presence of reinforcing fibers, whereas the right-hand images (post-exposure) illustrate microstructural transformations, including changes in pore wall integrity and the partial or complete loss of fibers. In several post-exposure images, reinforcing fibers are no longer visible, which is most likely attributable to thermal degradation or combustion during high-temperature exposure. The microstructural observations align with compositional findings, indicating that fiber type exerts a measurable influence on mesoscale porosity retention, interfacial cohesion, and the extent of thermally induced phase transformations, as further discussed in the oxide redistribution analysis.

The microstructure of the reference specimen without pores (Figure 16a) displayed the most compact and homogeneous morphology. The absence of any reinforcing fibers and the low overall porosity resulted in a dense, continuous alkali-activated hybrid binder matrix, serving as a baseline for evaluating fiber-induced modifications. In the case of the glass fiber-reinforced sample (HGP/G, Figure 16b), the fibers remained visible after simulated fire exposure, indicating that despite their relatively weak interfacial bonding with the alkali-activated hybrid binder matrix, they possess sufficient thermal stability to resist degradation at the applied temperature range. The basalt fiber composite (HGP/B, Figure 16c) exhibited numerous interconnected pores with diameters exceeding 1 mm. The presence of large, unevenly distributed pores reflects a foaming process lacking stability, a condition likely worsened by the omission of agents that promote structural uniformity. This instability resulted in areas where the foam collapsed locally, producing zones of uneven compaction. The resulting heterogeneity diminishes consistency in material performance and promotes higher rates of heat transfer, thereby reducing overall thermal insulation capability [108]. Bonding between the basalt fibers and the surrounding matrix was moderate; although some physical anchoring was retained, the extensive, interconnected porosity provided pathways for accelerated thermal ingress, weakening the reinforcing effect of the fibers. In the polypropylene fiber-reinforced hybrid (HGP/PP, Figure 16d), pre-exposure images reveal a high quantity of fibers well embedded in the foamed matrix, often coated with an alkali-activated hybrid binder. The majority of pores in this material were open, forming pathways through the matrix. After fire exposure, the fibers were no longer discernible. This aligns with the established thermal response of polypropylene, which softens and liquefies at around 170 °C well below the 190–260 °C range associated with peak vapor pressure in concrete [109]. Once melted, portions of the polymer infiltrate and are absorbed by the surrounding cementitious matrix [110]. At roughly 350 °C, the material undergoes complete combustion, yielding carbon dioxide and water vapor. The transient melting and combustion of PP fibers create additional open channels, which may facilitate vapor release but also reduce post-exposure mechanical cohesion. The merino wool fiber composite (HGP/M, Figure 16e) exhibited excellent fiber–matrix adhesion, as highlighted by the arrows in the micrographs. This strong interfacial bonding is likely due to the natural surface scales of wool fibers, which mechanically interlock with the alkali-activated hybrid binder, combined with possible chemical interactions between keratin and alkaline species in the matrix during curing. Such integration enhances stress transfer between fiber and matrix, improves crack-bridging capacity, and may contribute to maintaining integrity under thermal loading. Post-exposure images still show intact wool fibers, indicating that the fibers resisted complete combustion or charring. Wool fibers tend to form a char layer that acts as a thermal barrier, protecting the underlying structure. The observed pores were mostly spherical and uniform, with a significant proportion being open pores, which could aid in vapor release but also slightly reduce mechanical density. The coconut fiber composite (HGP/CO, Figure 16f) also demonstrated good integration with the foamed alkali-activated hybrid binder matrix. The rough surface of coconut fibers provides high mechanical interlock and strong adhesion. The pores in this sample were regular, spherical, and relatively small—features typically associated with favorable thermal insulation properties. However, despite this advantageous pore geometry, thermal endurance was limited: the thermal insulation threshold was maintained for 58 min, but a visible surface crack appeared on the unexposed side after approximately 40 min and progressively deepened. This suggests that while the pore structure effectively reduced heat transfer initially, differential thermal expansion between the fibers and the surrounding matrix, combined with moisture migration and possible fiber degradation, contributed to internal stress development and premature crack initiation.

The experimental results clearly indicate that, among all investigated composites, two fiber reinforcement types glass fibers (HGP/G) and merino wool fibers (HGP/M) exhibited particularly noteworthy performance in terms of fire resistance and microstructural stability. The merino wool–reinforced composite achieved the longest fire insulation retention time (83 min), as evidenced by the delayed temperature rise profile and the low color-difference gradient in the non-exposed layer, indicative of effective thermal diffusion attenuation. This behavior can be attributed to the high air-retention capacity and low thermal conductivity of wool, combined with excellent interfacial adhesion between the fibers and the alkali-activated hybrid binder matrix. The glass fiber–reinforced composite, despite limited interfacial bonding with the matrix and susceptibility to chemical corrosion, also demonstrated substantial thermal stability, maintaining a fire insulation duration of 57 min, with the fiber structure remaining intact post-exposure. Both reinforcement systems, due to their resistance to high-temperature degradation and potential to enhance thermal insulation performance, represent promising candidates for further research aimed at optimizing fiber–matrix integration and improving long-term fire protection efficiency.

## 4. Conclusions

This study demonstrated that fiber reinforcement critically influences the fire resistance and structural stability of foamed alkali-activated hybrid binders derived from recycled aluminosilicate precursors. Merino wool fibers (HGP/M) provided the longest insulation (83 min) due to a fine, interconnected pore network, while glass fibers (HGP/G) achieved the best balance of properties, combining moderate insulation (57 min) with minimal volumetric shrinkage (10.0%). Basalt (HGP/B) and polypropylene (HGP/PP) composites suffered from large, coalesced pores or polymer melting, limiting their performance, whereas coconut fiber (HGP/CO) showed early cracking. XRF analysis revealed temperature-driven redistribution of oxides, with refractory MgO and Al_2_O_3_ stabilizing the matrix and SiO_2_ forming glass phases. Colorimetric and XCT analyses confirmed that pore size, distribution, and interconnectivity—rather than porosity alone—govern thermal performance. Overall, wool fibers optimized thermal shielding, while glass fibers ensured dimensional stability, making HGP/G the most structurally robust formulation for sustainable, fire-safe construction.

Importantly, the demonstrated thermal stability, limited shrinkage, and controlled moisture behavior of the studied formulations also highlight their potential for additive manufacturing. The combination of colorimetric diagnostics and XCT-based pore characterization provides a framework for process monitoring and quality control in 3D-printed elements. Among the tested systems, the glass fiber–reinforced mixture (HGP/G) emerges as particularly promising for extrusion-based 3D printing, due to its low volumetric contraction and uniform structure, while the merino wool–reinforced mixture (HGP/M) offers advantages in thermal shielding. Taken together, these findings suggest that the developed alkali-activated hybrid binders can be feasibly adapted to 3D printing technologies, enabling the fabrication of sustainable, thermally resistant building components. The authors are currently extending this research by directly applying the optimized mixtures in 3D printing processes, and the results will be reported in forthcoming studies.

## Figures and Tables

**Table 1:** Composition and designations of an alkali-activated hybrid binder mixtures.

No.	Designation of the Mixture	w/s	Cement [%]	Sand [%]	Fly Ash [%]	Coal Slag [%]	Ground Bricks [%]	Coconut Fibers [%]	Basalt Fibers [%]	Glass Fibers [%]	Merino Fibers [%]	Polypropylene Fibers [%]
1	HGP/CO	0.22	27	37	5	15	15	0.5	-	-	-	-
2	HGP/B	0.22	27	37	5	15	15	-	0.5	-	-	-
3	HGP/G	0.22	27	37	5	15	15	-	-	0.5	-	-
4	HGP/M	0.24	27	37	5	15	15	-	-	-	0.5	-
5	HGP/PP	0.24	27	37	5	15	15	-	-	-	-	0.5
6	HGP	0.22	27	37	5	15	15	-	-	-	-	-

**Table 2:** Alkali-activated hybrid binder foam mixing protocol.

Process Number	Process Time [min]	Operation
1	2	Adding dry ingredients to the mixer and mixing them
2	15	Adding an activator and mixing
3	0.5	Mix integration verification
4	2	Preparing the concrete mass and mixing it at high speed (~500 rpm)
5	2	Alkali-activated hybrid binder production
6	1	Adding the remaining 50% of the total foam volume
7	2	Mixing the alkali-activated hybrid binder mixture and foam together
8	0.5	Mix integration verification
9	1	Adding the remaining part of the foam
10	2	Mixing the alkali-activated hybrid binder mixture and foam together

**Table 3:** The ΔE results depending on the mixture composition and the annealing temperature.

Temperature [°C]	HGP	HGP/G	HGP/B	HGP/PP	HGP/M	HGP/CO
200	2.21	5.99	5.00	7.16	6.84	5.57
400	7.52	5.34	9.31	5.75	5.98	8.83
600	6.92	11.42	10.68	12.81	5.38	20.85
800	9.70	12.76	11.41	6.52	6.05	8.55
1000	8.07	9.64	8.29	15.39	7.13	14.23

**Table 4:** ΔE of the samples after fire-exposure testing as a function of their composition and the identified layers.

Name of the Mixture	Layer Number	ΔE (Color Difference, CIE L*a*b*)
HGP	1	11.49
	2	7.61
	3	6.31
HGP/G	1	12.16
	2	12.13
	3	7.96
HGP/B	1	11.48
	2	12.19
	3	12.12
HGP/PP	1	12.29
	2	12.90
	3	4.26
HGP/M	1	7.50
	2	11.42
	3	3.97
HGP/CO	1	4.82
	2	19.69
	3	7.63

**Table 5:** Porosity analysis based on CT data as a function of their composition.

Series	HGP	HGP/G	HGP/B	HGP/PP	HGP/M	HGP/CO
Closed porosity [%]	6.29	0.18	0.01	0.01	0	0.36
Relative porosity [%]	11.79	41.44	55.19	66.86	74.37	61.77
Total volume sample [mm^3^]	42,875	42,875	42,875	42,913	42,875	42,875

## Data Availability

The original contributions presented in this study are included in the article. Further inquiries can be directed to the corresponding author.
